# Supplementary material for: The Systems Biology Research Tool: evolvable open-source software
Source: BMC Syst Biol. 2008 Jun 29;2:55. doi: 10.1186/1752-0509-2-55 (PMC2446383; doi:10.1186/1752-0509-2-55)
Supplement: Additional file 1 — SBRT Archive. An archive of the current version of the Systems Biology Research Tool. [file 1752-0509-2-55-S1.zip › sbrt-1.4.0/doc/developers_guide/api/sbrt/shell/io/EndOfFile.html]

EndOfFile


|  |  |  |
| --- | --- | --- |
| **Overview** \| **Package** \| **Class** \| **Use** \| **Tree** \| **Deprecated** \| **Index** \| **Help** | |  |
| **PREV CLASS**   **NEXT CLASS** | **FRAMES**    **NO FRAMES**     **All Classes** |  |
| SUMMARY: NESTED \| FIELD \| CONSTR \| METHOD | DETAIL: FIELD \| CONSTR \| METHOD |  |


---


## sbrt.shell.io Class EndOfFile

```
java.lang.Object
  sbrt.shell.io.EndOfFile
```

**All Implemented Interfaces:**: java.io.Serializable

---

``` public class EndOfFile extends java.lang.Object implements java.io.Serializable ```

This class is used to represent end-of-file (EOF).

**Author:**
:   This class was written and documented by Jeremiah Wright while in the
    Wagner lab.

**See Also:**: Serialized Form

---

**Constructor Summary**

`EndOfFile()`


| **Method Summary** | |
| --- | --- |
| `boolean` | `equals(java.lang.Object obj)`             Indicates if the provided object is equal to this end-of-file. |
| `static boolean` | `isEof(java.lang.Object obj)`             Indicates if the provided object is an instance of this class. |

**Methods inherited from class java.lang.Object**

`clone, finalize, getClass, hashCode, notify, notifyAll, toString, wait, wait, wait`

**Constructor Detail**

### EndOfFile

```
public EndOfFile()
```


**Method Detail**

### equals

```
public final boolean equals(java.lang.Object obj)
```

:   Indicates if the provided object is equal to this
    end-of-file.

    :   **Overrides:**: `equals` in class `java.lang.Object`
    :   **Parameters:**: `obj` - the reference object with which to compare. **Returns:**: `true` if the provided object is an instance of this class; `false` otherwise.

---


### isEof

```
public static final boolean isEof(java.lang.Object obj)
```

:   Indicates if the provided object is an instance of
    this class.

    :   **Parameters:**: `obj` - the reference object with which to compare. **Returns:**: `true` if the provided object is an instance of this class; `false` otherwise.


---


|  |  |  |
| --- | --- | --- |
| **Overview** \| **Package** \| **Class** \| **Use** \| **Tree** \| **Deprecated** \| **Index** \| **Help** | |  |
| **PREV CLASS**   **NEXT CLASS** | **FRAMES**    **NO FRAMES**     **All Classes** |  |
| SUMMARY: NESTED \| FIELD \| CONSTR \| METHOD | DETAIL: FIELD \| CONSTR \| METHOD |  |


---
